# Maternal asthma and the role of stress, sensitization, and lung function on pregnancy outcomes: MAESTRO cohort study

**DOI:** 10.1016/j.jacig.2026.100683

**Published:** 2026-03-19

**Authors:** Gustaf Rejnö, Cecilia Lundholm, Bronwyn K. Brew, Emma Caffrey Osvald, Catarina Almqvist

**Affiliations:** a Department of Medical Epidemiology and Biostatistics, Karolinska Institutet, Stockholm, Sweden; b Obstetrics and Gynaecology Unit, Södersjukhuset, Stockholm, Sweden; c School of Medicine and Public Health, University of Newcastle, Newcastle, Australia; d National Perinatal Epidemiology and Statistics Unit, Centre for Big Data Research in Health, University of New South Wales, Sydney, Australia; e Pediatric Allergy and Pulmonology Unit at Astrid Lindgren Children’s Hospital, Karolinska University Hospital, Stockholm, Sweden

**Keywords:** Asthma, pregnancy, epidemiology, spirometry, stress, allergy

## Abstract

**Background:**

Asthma is associated with adverse pregnancy and delivery outcomes. Stress, IgE sensitization, and lung function may contribute to these associations.

**Objective:**

We investigated the effect of allergy and stress on the association between maternal asthma and adverse outcomes; and examined the association between lung function and adverse perinatal outcomes.

**Methods:**

In the MAESTRO cohort of pregnant women recruited from urban antenatal clinics 2011-16, 1511 singleton pregnancies were followed to delivery (3.5% preterm), and 242 underwent spirometry. Asthma diagnosis and medication from national registers were linked to cohort data. Associations between allergic and nonallergic asthma (with and without IgE sensitization ≥ 0.35 kU_A_/L to airborne allergens) and perinatal outcomes were estimated by regression analyses. *Z* scores for forced expiratory volume in 1 second (FEV_1_), forced vital capacity (FVC), and FEV_1_/FVC ratio were used as exposures in secondary analyses. All analyses were adjusted for maternal stress on the bases of questionnaires, diagnoses, and medications.

**Results:**

Eighteen percent (n = 293) of women had asthma; 60% of these had allergic asthma. Compared to women without asthma, allergic asthma was associated with reduced mean gestational age by 0.23 weeks (95% confidence interval, −0.44, −0.03). No other adverse perinatal associations were found for allergic asthma, and none was found for nonallergic asthma. Stress did not appear to affect the associations. Of women who underwent spirometry, 89% (n = 215) had normal lung function, and we detected no significant associations between lung function and adverse outcomes.

**Conclusion:**

Allergic asthma is associated with slightly lower gestational age. In women with normal lung function ranges, there is likely no association with adverse perinatal outcomes.

Asthma in the pregnant woman is generally associated with several adverse pregnancy and delivery outcomes, and poor asthma control especially seems to increase the risk of hypertension in pregnancy, preterm delivery, and baby small for gestational age.^1^, ^2^, ^3^ It has also been shown that the association between asthma during pregnancy and adverse pregnancy and perinatal outcomes are independent of family factors such as shared genes and environment, suggesting a causal effect.^4^

Asthma is a heterogenous disease, with the two major phenotypes being allergic (sensitized) and nonallergic (nonsensitized) asthma. In allergic asthma, airway inflammation is initiated by IgE and driven by mast cells and eosinophils. In nonallergic asthma, airway inflammation occurs by proinflammatory cytokines released by immune cells, irrespective of IgE, or by nonimmunologic stimuli.^5^ Little is known about pregnancy outcomes among women with allergic asthma. A recent meta-analysis about neonatal outcomes in pregnant women with asthma noted that studies to date have not distinguished allergic from nonallergic asthma.^6^

Asthma affects lung function and has a complex impact on health.^7^ Reduced lung function is associated with several conditions, including cardiovascular diseases as well as obstructive and restrictive lung diseases.^8^ Jensen et al^9^ showed that in women without asthma, forced vital capacity (FVC) and forced expiratory volume in 1 second (FEV_1_) declined during pregnancy, whereas the FEV_1_/FVC ratio was unaffected. However, in women with asthma, FVC declined during pregnancy, FEV_1_ remained stable, and FEV_1_/FVC increased. Only a few studies have examined the associations between lung function in women with and without asthma and perinatal outcomes, and more studies are needed.

Previous studies have reported that stress and anxiety during pregnancy increase the risk of adverse pregnancy outcomes in women with asthma, including baby born at a shorter gestational age and with lower birth weight, independent of familial factors.^10^ Although this work was conducted using national health register data, a clinical study with better-defined asthma phenotypes (based on biomarkers and lung function) can further improve measurement accuracy and allow more in-depth study. Furthermore, little is known about how stress during pregnancy may affect lung function in pregnant women with asthma, although the association between asthma and anxiety/depression^11^^,^^12^ indicates that not only maternal asthma but also maternal stress should be addressed to optimize care of pregnant women.

The aim of this study was to examine the associations between maternal asthma (both allergic and nonallergic) with perinatal outcomes as well as maternal lung function and the association with perinatal outcomes. Also, we want to assess whether maternal stress plays a role in these associations.

## Methods

For this prospective cohort study, we used the clinical Maternal Asthma Events, Stress and Offspring (MAESTRO) cohort, in which 1693 pregnant women were recruited from 8 antenatal clinics in the Stockholm area between 2011 and 2016.^13^ At their first antenatal visit in the first trimester of pregnancy, all women visiting these clinics were invited to participate. Women who did not understand Swedish were excluded because all questionnaires were in Swedish.

At recruitment, blood from each participant was collected for analysis of IgE levels to airborne allergens. Serum was analyzed by Phadiatop (Thermo Fisher Scientific, Uppsala, Sweden), which is a screening test for common airborne allergens (timothy, birch, mugwort, cat, dog, horse, house dust mites [*Dermatophagoides pteronyssinus* and *farina*], and mold *[Cladosporium herbarum]*). Sera that tested positive in Phadiatop (≥0.35 kU_A_/L) were subsequently analyzed for IgE antibodies to the single allergens listed above (ImmunoCAP System, Thermo Fisher Scientific). Each participant was asked to complete a questionnaire including questions on background factors, their asthma status, the 10-item Perceived Stress Scale (PSS-10)^14^, ^15^, ^16^ and a depression questionnaire (CES-D).^17^ Women were followed during their pregnancy for blood and saliva sample collection, questionnaires, and lung function measurements.

In addition to the questionnaires we collected, we also retrieved records from participants’ midwife and hospital visits.

The whole cohort was linked to 3 Swedish national health registers on the basis of the personal identity number: the Medical Birth Register with data on almost all births in Sweden from 1973;^18^ the Swedish Prescribed Drugs Register with data on all prescribed dispensed drugs since July 2005;^19^ and the National Patient Register with all inpatient and outpatient specialist care diagnoses since 2001.^20^

On the basis of replies to the questionnaire administered at gestational weeks 10-12 on asthma or asthma-like symptoms, a subset of women with and without asthma with singleton pregnancies were invited to undergo spirometry during pregnancy weeks 28-32. The size of the required subpopulation was determined by power calculations (a 10% difference in FVC and a 10% difference in FEV_1_ between women with and without asthma, significance level = .05, power = 80%). The lung function measurements were performed by a trained research nurse with the SpiroStar Pro v2.3.1 system (Medikro Spirometry Software, Kuopio, Finland) according to American Thoracic Society/European Respiratory Society guidelines.^21^^,^^22^

### Exposures

The main exposure was asthma in the year before pregnancy until delivery; asthma, either self-reported (questionnaires) or physician diagnosed according to a validated algorithm;^23^ an asthma diagnosis in the National Patient Register (International Classification of Diseases, 10th edition [ICD-10], codes J45, J46); and/or at least two dispenses of asthma medication (Anatomical Therapeutic Chemical classification [ATC] code R03) in the Swedish Prescribed Drug Register. Sensitization was defined as allergen-specific IgE ≥ 0.35 kU_A_/L to any of the tested airborne allergens. This left us with 3 groups: (1) women without asthma (reference), (2) women with asthma but no IgE sensitization (nonallergic asthma), and (3) women with positive IgE sensitization (allergic asthma). In the analysis with allergic or nonallergic asthma as exposures, we only included participants with data on IgE status.

For the secondary analysis on lung function, FEV_1_ and FVC values measured before and after bronchodilator therapy (terbutaline) in liters^22^ were converted to *z* scores based on the Global Lung Function Initiative,^24^ which accounts for sex, age, height, and ethnicity.

### Outcomes

The perinatal outcomes included the following: hypertensive disorders in pregnancy (including preeclampsia); mode of delivery (vaginal noninstrumental, vaginal instrumental, caesarean section [elective/emergency]); respiratory distress in the child (ICD-10 codes P22-P28); and *z* scores for gestational age and birth weight. We also used *z* scores for birth weight by gestational age calculated by using the mean birth weight by gestational age and standard deviation according to Marsal et al.^25^ We were unable to study the association with hypertension in pregnancy in the secondary analyses on lung function because there were too few individuals with that outcome.

All data on the outcomes were collected from the medical records and the Medical Birth Register.

### Covariates

**Supplementary Fig E1:**
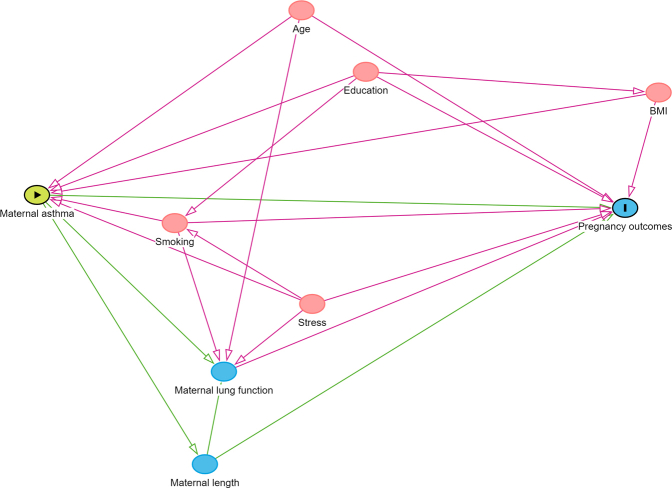


We used a directed acyclic graph^26^^,^^27^ to identify important covariates based on subject-specific knowledge (see Fig E1 in this article’s Online Repository available at www.jaci-global.org). The possible confounders identified for adjustment in models included: maternal stress, maternal age at delivery (continuous), maternal height (continuous), and body mass index (BMI), collected from the questionnaires, medical records, and the Medical Birth Register. We also identified maternal education as a possible confounder, but because this variable was gathered from the questionnaires with a response rate of around 70%, we were unable to adjust for this in the whole cohort because of missing data. Maternal education was included as a covariate in the secondary analyses with lung function as our exposure because all participants in the subcohort completed the questionnaire.

Our maternal stress variable was a composite measure defined as either a CES-D score of ≥16 or medication for anxiety/depression from the year before pregnancy until delivery (at least 2 dispenses with an interval of more than 2 weeks of ATC codes N05B or N06A) or anxiety/depression diagnosis (ICD-10 codes F30-F34, F38-F42, F44, F45, or F48). We also added information from the PSS-10 questionnaire, and because there is no predefined cutoff value for PSS-10 we chose a cutoff of above the 80th percentile (18 points) to harmonize with the CES-D score of ≥16, which is also on the 80th percentile for this cohort.

### Statistical analyses

We first estimated crude and adjusted odds ratios and β coefficients with 95% confidence intervals (CIs) for the associations between our exposures and studied outcomes with logistic and linear regression for the dichotomous and continuous outcomes, respectively. Because in the MAESTRO cohort a few women contributed more than one pregnancy to the dataset, we used the sandwich estimator for standard errors.

To assess the effect of maternal education in the primary analyses, we performed a sensitivity analysis in the subgroup of women who answered the questionnaire, both with and without adjustment for education.

All analyses were performed by Stata IC v18 software (StataCorp, College Station, Tex).

### Ethics

All participants signed a written consent to the study before enrollment. All data were pseudonymized before analyses, and all results are presented at the group level only. The study was approved by the regional ethical review board in Stockholm.

## Results

**Fig 1:**
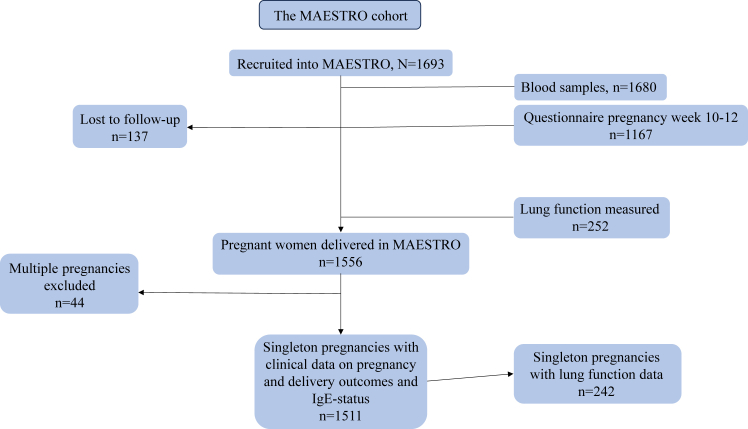
MAESTRO cohort description.

Of the original cohort of 1693 pregnancies, 1556 pregnancies were followed to delivery, and the final study cohort consisted of 1511 singleton pregnancies with information on IgE sensitization (Phadiatop) and clinical data on pregnancy and delivery outcomes (Fig 1).

Of these, 1218 (81%) were women without asthma; of those with asthma, 118 (8%) had nonallergic and 175 (12%) allergic asthma. In general, women with asthma had a slightly higher BMI and maternal stress was more common in women with asthma compared to women without—and stress was especially high in women with nonallergic asthma (Table I).

**Table I:** Characteristics of MAESTRO cohort

Outcome/covariate	Total	No asthma	IgE-negative asthma	IgE-positive asthma
No. of subjects	1511	1218	118	175
Maternal age				
<29 years	385 (25.5)	310 (25.5)	28 (23.7)	47 (26.9)
29-32 years	437 (28.9)	357 (29.3)	33 (28.0)	47 (26.9)
>32 years	689 (45.6)	551 (45.2)	57 (48.3)	81 (46.3)
BMI				
≤25 kg/m^2^	1117 (73.9)	925 (76.0)	75 (63.6)	120 (68.6)
>25-30 kg/m^2^	239 (15.8)	179 (14.7)	29 (24.6)	28 (16.0)
>30 kg/m^2^	56 (3.7)	37 (3.0)	7 (5.9)	12 (6.9)
Missing	99 (6.6)	77 (6.3)	7 (5.9)	15 (8.6)
Smoking				
Yes	12 (0.8)	7 (0.6)	4 (3.4)	1 (0.6)
Missing	7 (0.5)	6 (0.5)	0	1 (0.6)
Education				
0-9 years	7 (0.5)	6 (0.5)	0	1 (0.6)
10-12 years	140 (9.3)	101 (8.3)	15 (12.7)	24 (13.7)
≥13 years	919 (60.8)	746 (61.2)	72 (61.0)	101 (57.7)
Missing	445 (29.5)	365 (30.0)	31 (26.3)	49 (28.0)
Cohabitation	1461 (96.7)	1175 (96.5)	116 (98.3)	170 (97.1)
IgE				
Positive	504 (33.4)	329 (27.0)	0	175 (100.0)
Missing	75 (5.0)	75 (6.2)	0	0
Maternal distress∗	346 (22.9)	260 (21.3)	42 (35.6)	44 (25.1)
PSS-10 > 18	163 (10.8)	121 (9.9)	20 (16.9)	22 (12.6)
CES-D ≥ 16	206 (13.6)	155 (12.7)	27 (22.9)	24 (13.7)
Anxiety/depression				
Medication	98 (6.5)	76 (6.2)	11 (9.3)	11 (6.3)
Diagnosis	48 (3.2)	39 (3.2)	5 (4.2)	4 (2.3)
Sex				
Male	780 (51.6)	617 (50.7)	63 (53.4)	100 (57.1)
Female	731 (48.4)	601 (49.3)	55 (46.6)	75 (42.9)

Data are presented as nos. (%).

∗ Maternal PSS-10 score above 80th percentile (equals a score of 18) around gestational week 10 and/or CES-D score ≥ 16 around gestational week 10 and/or anxiety/depression medication from 1 year before pregnancy until delivery and/or anxiety/depression diagnosis from 1 year before pregnancy until delivery.

Data on maternal education, retrieved from questionnaires, had the largest proportion missing in the group of women without asthma (30.0%). Background data on women who completed and did not complete the early pregnancy questionnaire are listed in Table E1 in the Online Repository available at www.jaci-global.org. Those who did not complete the questionnaire were younger, were more likely to smoke (although only a very few in total smoked), and were less likely to cohabitate. They were also, to a slightly higher extent, more often prescribed medication to treat or received a diagnosis of anxiety and/or depression.

Among individuals who filled out the questionnaire, maternal education level was slightly lower in women with allergic asthma; missing data did not differ between the groups.

Most women had well-controlled asthma, and none had disease treated with monoclonal antibodies (ATC codes R03DX) or allergen immunotherapy (ATC code V01). However, 4 individuals experienced an exacerbation in the year before delivery (defined as a prescription for oral corticosteroids and/or emergency visit or admission for asthma). As a proxy for uncontrolled asthma, 6 individuals had ≥5 dispensings of short-acting β-agonists in the year before delivery (data not shown).

Of the 481 women who were invited for spirometry, 242 women underwent the examination, and 82 reported an asthma diagnosis in the questionnaires (34%). Of these, 52 had positive Phadiatop results. No differences in FEV_1_, FVC, or FEV_1_/FVC were observed between groups (see Table E2 in the Online Repository available at www.jaci-global.org). Of all women, 89% (n = 215) had normal lung function (defined as FEV_1_/FVC > 0.70 and FEV_1_ and FVC above 80% of the predicted value) before administration of bronchodilator therapy, and 93% (n = 225) showed normal ranges after bronchodilator therapy (data not shown in tables).

Mean gestational age at birth in women with nonallergic asthma was 40.1 weeks and in women with allergic asthma was 39.7 weeks (see Table E3 in the Online Repository available at www.jaci-global.org). In total, 53 pregnancies (3.5%) ended before term (<37 weeks’ gestation).

**Fig 2:**
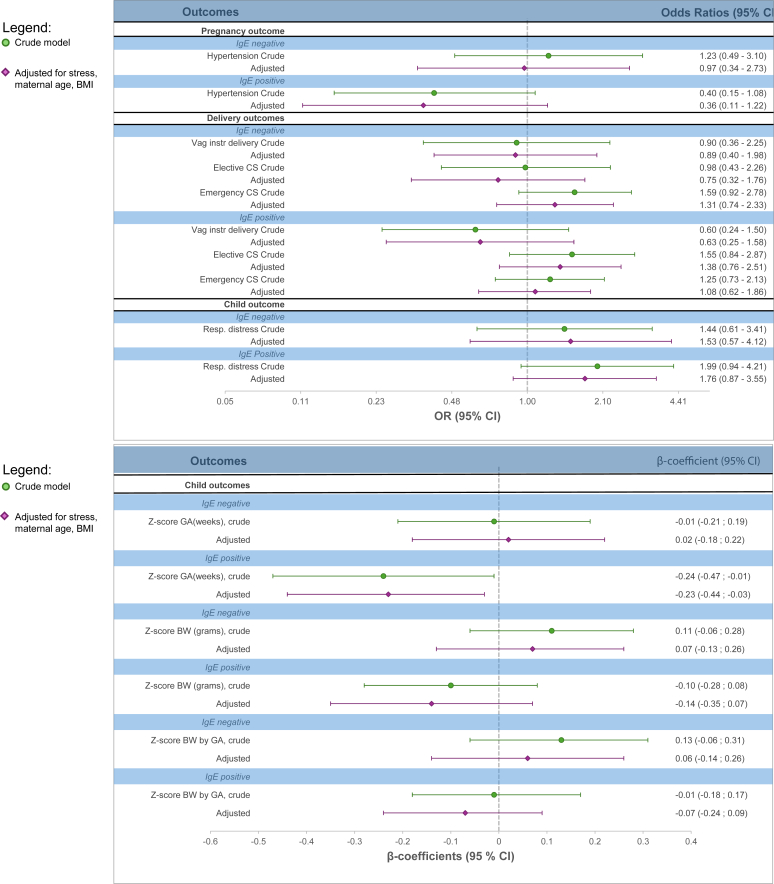
Nonallergic and allergic asthma and odds ratios and β coefficients with 95% CIs for adverse pregnancy and perinatal outcomes. Respiratory distress reported according to ICD-10 diagnoses P22-P28 in newborn child. Adjusted model adjusted for maternal stress, age, and BMI.

No statistically significant associations were observed for allergic or nonallergic asthma and perinatal outcomes compared to no asthma, except for a lower mean gestational age by 0.23 weeks (95% CI, −0.44, −0.03), among mothers with allergic asthma (Fig 2).

However, we observed point estimates indicating several adverse outcomes for pregnancies of women with allergic asthma, such as respiratory distress of the child (odds ratio = 1.96; 95% CI, 1.05, 3.64, in the model adjusted for stress only; and odds ratio = 1.76; 95% CI, 0.87, 3.55, in the model adjusted for maternal stress, age, and BMI) (Table E3, Fig 2).

**Fig 3:**
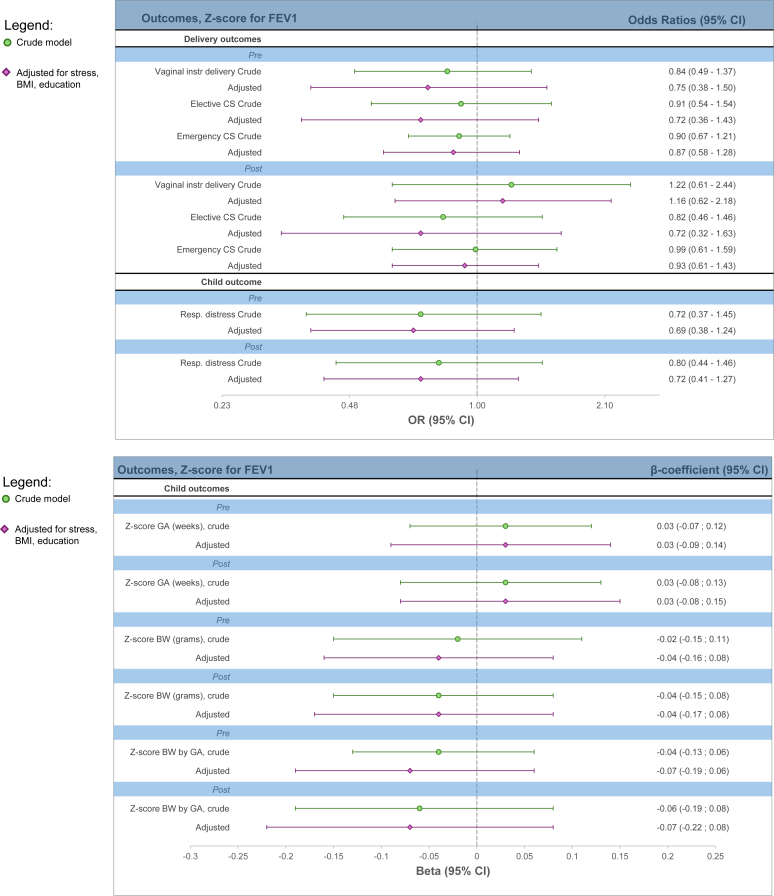

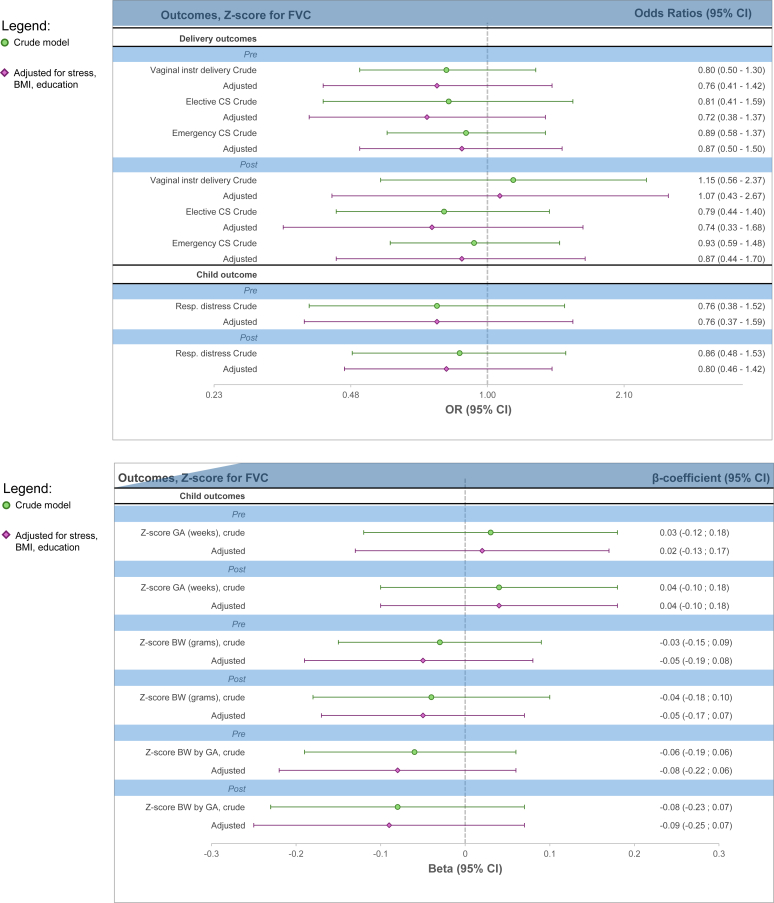

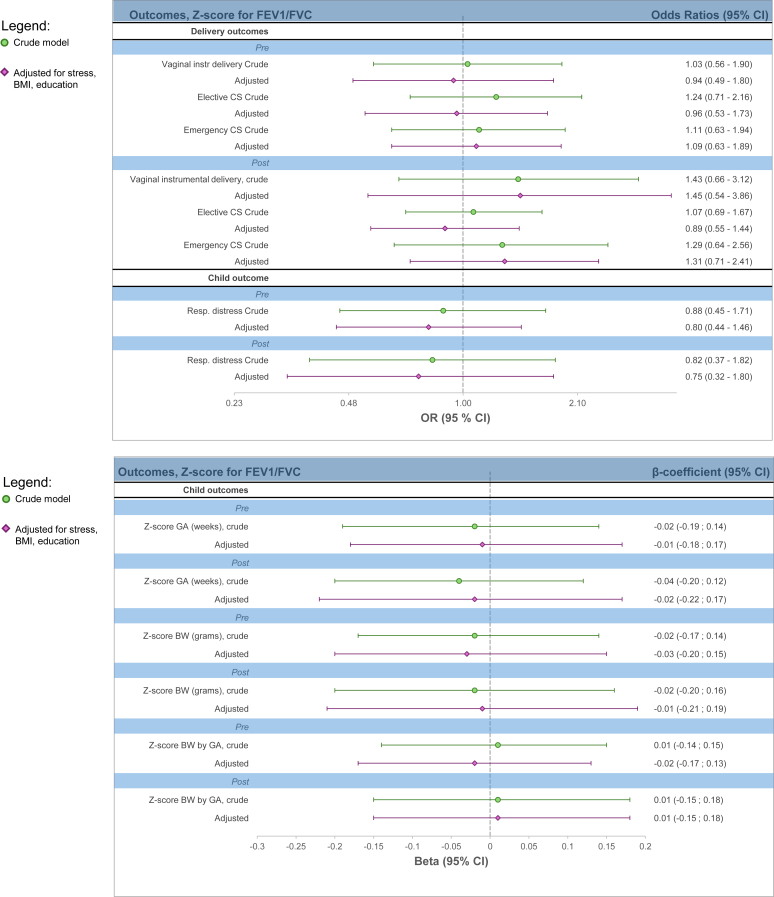
*Z* score for lung function before and after bronchodilation therapy. Respiratory distress reported according to ICD-10 diagnoses P22-P28 in newborn child. Adjusted model adjusted for maternal stress, BMI, and education.

In the secondary analyses of lung function, there were no observed significant associations with the perinatal outcomes. For instance, in the prebronchodilation analysis, the point estimate indicated that a 1 *z* score higher FEV_1_ corresponded to a 0.03 higher *z* score for gestational age (β = 0.03; 95% CI, −0.09, 0.14) (see Table E4 in the Online Repository available at www.jaci-global.org; and Fig 3).

In the sensitivity analyses, with data only from questionnaires, where we adjusted additionally for maternal education, the estimates were similar to the original analyses, and the changes when additionally adjusting for education were minor (see Table E5 in the Online Repository available at www.jaci-global.org).

## Discussion

In this cohort study of 1511 pregnant women with singletons who were followed throughout pregnancy and delivery, we found that women with allergic asthma gave birth earlier compared to women without asthma, irrespective of maternal stress. In women with nonallergic asthma, we did not detect any statistically significant differences compared to women without asthma.

There was no difference between the groups for birth weight by gestational age, suggesting that asthma could drive shorter gestational age, which explains the slightly lower birth weight observed among the infants born to women with allergic asthma. The mechanism behind this is unknown, but it could be related to an unbalance in the T_H_1/T_H_2 distribution leading to more inflammation and less fetal tolerance.^28^ The results here with lower gestational age in women with allergic asthma are similar to the results in a study by Bartha et al^29^ that reported that women with respiratory allergy were at higher risk of prematurity and low birth weight; however, in that study, the effects were suggested to be mediated by sterility, *in vitro* fertilization, and multiple gestations; and in a Japanese study of 97,000 women, a history of maternal allergic disease was found to be associated with threatened preterm birth but not premature birth.^30^ We defined allergic asthma as sensitization to allergen-specific IgE, so many of those individuals would also have allergic rhinitis, which is part of the allergic manifestation.

Even though our results only show a minor decrease in gestational age of infants born to women with allergic asthma, a slightly lower gestational age in term pregnancies is associated with adverse outcomes in the child, such as asthma^31^ or autism spectrum disorders.^32^

In the subanalysis on lung function during pregnancy, in a cohort with average normal lung function ranges, we were not able to detect any statistically significant associations between lung function, measured as FEV_1_, FVC, and FEV_1_/FVC and adverse pregnancy or perinatal outcomes.

We had a high prevalence of stress measured as perceived stress as well as anxiety or depression in our cohort. In a study from Bedolla-Barajas et al^33^ from 2021, anxiety and depression were present in more than 50% of patients with asthma, and levels of anxiety were highest in patients with nonallergic asthma, which is in line with our findings.

In a previous population-based study that used the same register-based algorithm measuring stress as anxiety and depression (prevalence, 5.9%), we found associations between maternal stress and adverse pregnancy outcomes such as preeclampsia in the mother, emergency caesarean section, and baby small for gestational age.^10^ In the current study, where we studied maternal stress as a confounder, a large proportion of the cohort (eg, 41% of women with allergic asthma who completed the questionnaire) reported stress. We used a combination of different sources including data from questionnaires, possibly widening the stress definition from what the previous study had used by including mild stress—that is, stress that is not severe enough to warrant a clinical diagnosis. So although stress may affect pregnancy and delivery outcomes,^34^ in our study, we were unable to determine a confounding effect.

This study has several strengths, including an asthma-rich cohort with detailed data on covariates such as stress, lung function data, and information on sensitization during pregnancy, with participants followed prospectively rather than retrospectively, thus minimizing recall and measurement bias. There are also some limitations to this study. In a relatively small cohort like this, there are low numbers for some rare outcomes, such as hypertension in pregnancy and instrumental delivery, which make them difficult to study. The majority of those who had lung function measured had lung volumes within the normal range; this, together with the low smoking rates and high educational attainment, suggest that the cohort may not be representative of the general population, so our study may not have been able to detect the effects of extremes—for example, if low lung function drives worse outcomes. However, within the range of normal lung function, we did not see a linear effect, which means that for most of the population, including women with asthma, our results contribute to the knowledge that lung function in pregnancy is not associated with adverse perinatal outcomes, regardless of stress. This highlights the importance of good asthma control during pregnancy. The small size of the cohort also limited the study of more severe or uncontrolled asthma and the association with adverse outcomes.

To conclude, allergic asthma is associated with slightly shorter gestational age, regardless of maternal stress, which could be clinically relevant in the setting of a more premature infant. We found no evidence of nonallergic asthma’s being associated with adverse pregnancy outcomes. In an asthma-rich population of pregnant women mostly within normal lung function ranges, we found no evidence of an association with adverse perinatal outcomes.

## Disclosure statement

Financial support was provided from the 10.13039/501100004359Swedish Research Council (grants 2018-02640 and 2023-02327), the Strategic Research Program in Epidemiology at Karolinska Institutet, the 10.13039/501100003793Swedish Heart-Lung Foundation (grants 20210416 and 20240974), the Swedish Asthma and Allergy Association Research Fund (grant 2024-0010), and the Foundation Frimurare Barnhuset in Stockholm.

Data availability statement: The data that support the findings of this study are available on request from the corresponding author. The data are not publicly available because of privacy or ethical restrictions.

Disclosure of potential conflict of interest: The authors declare that they have no relevant conflicts of interest.

## Supplementary data

Supplementary Table E1

Supplementary Table E2

Supplementary Table E3

Supplementary Table E4

Supplementary Table E5
