# Supplementary material for: Maternal asthma and the role of stress, sensitization, and lung function on pregnancy outcomes: MAESTRO cohort study
Source: J Allergy Clin Immunol Glob. 2026 Mar 19;5(3):100683. doi: 10.1016/j.jacig.2026.100683 (PMC13087686; doi:10.1016/j.jacig.2026.100683)
Supplement: Supplementary Table E1 [file mmc1.docx]

**Supplemental Table 1. Background data on participants that completed and did not complete questionnaire in early pregnancy.**

| **Outcomes/covariates** | **Total questionnaire** | **Total no questionnaire** | **No asthma questionnaire** | **No asthma, no questionnaire** | **Asthma, IgE neg, questionnaire** | **Asthma, IgE neg, no questionnaire** | **Asthma, IgE pos, questionnaire** | **Asthma, IgE pos, no questionnaire** |
| --- | --- | --- | --- | --- | --- | --- | --- | --- |
| N | 1080 | 431 | 860 | 358 | 89 | 29 | 131 | 44 |
| Maternal age, years <29 | 249 (23.1) | 136 (31.6) | 195(22.7) | 115 (32.1) | 19 (21.4) | 9 (31.0) | 35 (26.7) | 12 (27.3) |
| 29-32 | 327 (30.3) | 110 (25.5) | 264 (30.7) | 93 (26.0) | 28 (31.3) | 5 (17.2) | 35 (26.7) | 12 (27.3) |
| >32 | 504 (46.7) | 185 (42.9) | 401 (46.6) | 150 (41.9) | 42 (47.2) | 15 (51.7) | 61 (46.6) | 20 (45.5) |
| BMI, kg/m^2^ ≤ 25 | 797 (73.8) | 323 (74.9) | 652 (75.8) | 273 (76.3) | 53 (59.6) | 22 (75.9) | 92 (70.2) | 28 (63.6) |
| >25-30 | 171 (15.8) | 65 (15.1) | 126 (14.7) | 53 (14.8) | 24 (27.0) | 5 (17.2) | 21 (16.0) | 7 (15.9) |
| >30 | 35 (3.2) | 21 (4.9) | 23 (2.7) | 14 (3.9) | 6 (6.7) | 1 (3.4) | 6 (4.6) | 6 (13.6) |
| missing | 77 (7.1) | 22 (5.1) | 59 (6.7) | 18 (5.0) | 6 (6.7) | 1 (3.4) | 12 (9.2) | 3 (6.8) |
| Smoking | 5 (0.5) | 7 (1.6) | 3 (0.4) | 4 (1.1) | 2 (2.3) | 2 (6.9) | 0 (0.0) | 1 (2.3) |
| Smoking Missing | 2 (0.2) | 5 (1.2) | 1 (0.1) | 5 (1.4) | 0 (0.0) | 0 (0.0) | 1 (0.8) | 0 (0.0) |
| Education 0-9 years | 7 (0.7) | 0 (0.0) | 6 (0.7) | 0 (0.0) | 0 (0.0) | 0 (0.0) | 1 (0.8) | 0 (0.0) |
| Education 10-12 years | 140 (13.0) | 0 (0.0) | 101 (11.7) | 0 (0.0) | 15 (16.9) | 0 (0.0) | 24 (18.3) | 0 (0.0) |
| Education ≥ 13 years | 919 (85.1) | 0 (0.0) | 746 (86.7) | 0 (0.0) | 72 (80.9) | 0 (0.0) | 101 (77.1) | 0 (0.0) |
| Education missing | 14 (1.3) | 431 (100.0) | 7 (0.8) | 358 (100.0) | 2 (2.3) | 29 (100.0) | 5 (3.8) | 44 (100.0) |
| Cohabitation | 1059 (98.1) | 402 (93.3) | 843 (98.0) | 332 (92.7) | 88 (98.9) | 28 (96.6) | 128 (97.7) | 42 (95.5) |
| IgE positive | 355 (32.9) | 149 (34.6) | 224 (26.1) | 105 (29.3) | 0 (0.0) | 0 (0.0) | 131 (100.0) | 44 (100.0) |
| IgE missing | 46 (4.3) | 29 (6.7) | 46 (5.4) | 29 (8.1) | 0 (0.0) | 0 (0.0) | 0 (0.0) | 0 (0.0) |
| Maternal distress* | 305 (28.2) | 41 (9.5) | 226 (26.3) | 34 (9.5) | 37 (41.6) | 5 (17.2) | 42 (32.1) | 2 (4.5) |
| - PSS-10 > 18 | 163 (15.1) | 0 (0.0) | 121 (14.1) | 0 (0.0) | 20 (22.5) | 0 (0.0) | 22 (16.8) | 0 (0.0) |
| - CES-D ≥ 16 | 206 (19.1) | 0 (0.0) | 155 (18.0) | 0 (0.0) | 27 (30.3) | 0 (0.0) | 24 (18.3) | 0 (0.0) |
| - Anxiety/depr med | 64 (5.9) | 34 (7.9) | 49 (5.7) | 27 (7.5) | 6 (6.7) | 5 (17.2) | 9 (6.9) | 2 (4.5) |
| - Anxiety/depr diag | 27 (2.5) | 21 (4.9) | 21 (2.4) | 18 (5.0) | 3 (3.4) | 2 (6.9) | 3 (2.3) | 1 (2.3) |
| Male | 551 (51.0) | 229 (53.1) | 433 (50.3) | 184 (51.4) | 45 (50.6) | 18 (62.1) | 65 (53.7) | 27 (61.4) |
| Female | 529 (49.0) | 202 (46.9) | 427 (49.7) | 174 (48.6) | 44 (49.4) | 11 (37.9) | 56 (46.3) | 17 (38.6) |

*Maternal PSS-10-score above 80^th^ percentile (18) around gestational week 10 and/or CESD-score ≥ 16 around gestational week 10 and/or Anxiety/depression medication from 1 year before pregnancy until delivery and/or Anxiety/depression diagnose from 1 year before pregnancy until delivery
