# Supplementary material for: Maternal asthma and the role of stress, sensitization, and lung function on pregnancy outcomes: MAESTRO cohort study
Source: J Allergy Clin Immunol Glob. 2026 Mar 19;5(3):100683. doi: 10.1016/j.jacig.2026.100683 (PMC13087686; doi:10.1016/j.jacig.2026.100683)
Supplement: Supplementary Table E2 [file mmc2.docx]

**Supplemental Table 2. Mean spirometry values and standard deviation in women with and without asthma**

| **Outcomes/covariates** | **Total** | **No asthma** | **Asthma IgE neg** | **Asthma IgE pos** |
| --- | --- | --- | --- | --- |
| N | 242** | 152 | 30 | 52 |
| Absolute (l) FEV_1_ pre | 3.32 (0.54) | 3.34 (0.51) | 3.39 (0.68) | 3.27 (0.52) |
| Absolute (liters) FVC pre | 4.13 (0.65) | 4.12 (0.63) | 4.30 (0.82) | 4.11 (0.60) |
| Absolute (l) FEV1 post | 3.41 (0.51) | 3.42 (0.55) | 3.44 (0.47) | 3.37 (0.42) |
| Absolute (liters) FVC post | 4.11 (0.63) | 4.10 (0.66) | 4.27 (0.60) | 4.10 (0.53) |
| Relative (%) FEV_1_ pre | 104 (14) | 104 (13) | 103 (16) | 105 (14) |
| Relative (%) FVC pre | 109 (14) | 109 (13) | 110 (16) | 111 (13) |
| Relative (%) FEV_1_/FVC pre | 95 (6) | 96 (6) | 94 (7) | 94 (7) |
| Relative (%) FEV_1_ post | 107 (13) | 107 (13) | 104 (12) | 108 (10) |
| Relative (%) FVC post | 108 (13) | 108 (14) | 108 (12) | 111 (11) |
| Relative (%) FEV_1_/FVC post | 98 (5) | 99 (5) | 96 (6) | 97 (5) |

*FEV1 = Forced expiratory volume (liters) in one second, FVC = forced vital capacity in liters. Pre = pre broncho dilator, post = post broncho dilator
**Including 8 participants with missing asthma status
