# Supplementary material for: Maternal asthma and the role of stress, sensitization, and lung function on pregnancy outcomes: MAESTRO cohort study
Source: J Allergy Clin Immunol Glob. 2026 Mar 19;5(3):100683. doi: 10.1016/j.jacig.2026.100683 (PMC13087686; doi:10.1016/j.jacig.2026.100683)
Supplement: Supplementary Table E3 [file mmc3.docx]

**Supplemental table 3.** Non-allergic and allergic asthma and odds ratios and beta-coefficients for adverse pregnancy and perinatal outcomes.

|  | w/o asthma | Asthma, IgE negative | | | | Asthma, IgE positive | | | |
| --- | --- | --- | --- | --- | --- | --- | --- | --- | --- |
| Exposure groups |  | **n(%)** | **Crude OR/β-coeff** | **Adj* OR/β-coeff** | **Adj** OR/β-coeff** | **n(%)** | **Crude OR/β-coeff** | **Adj* OR/β-coeff** | **Adj** OR/β-coeff** |
|  | n=1218 | n=118 | (95% CI) | (95% CI) | (95% CI) | n=175 | (95% CI) | (95% CI) | (95% CI) |
|  |  |  |  |  |  |  |  |  |  |
| *Maternal outcomes* |  |  |  |  |  |  |  |  |  |
| *Hypertension in pregnancy* | 68 (5.6) | 8 (6.8) | 1.23 (0.49 – 3.10) | 1.22 (0.51 – 2.90) | 0.97 (0.34 – 2.73) | 4 (2.3) | 0.40 (0.15 – 1.08) | 0.39 (0.11 – 1.39) | 0.36 (0.11 – 1.22) |
| *Delivery Mode* |  |  |  |  |  |  |  |  |  |
| *Vaginal non-instrumental delivery* | 909 (74.6) | 85 (72.0) | Ref. | Ref. | Ref. | 128 (73.1) | Ref. | Ref. | Ref. |
| *Vaginal instrumental delivery* | 83 (6.8) | 7 (5.9) | 0.90 (0.36 – 2.25) | 0.88 (0.35 – 2.22) | 0.89 (0.40 – 1.98) | 7 (4.0) | 0.60 (0.24 – 1.50) | 0.60 (0.24 – 1.48) | 0.63 (0.25 – 1.58) |
| *Elective CS* | 87 (7.1) | 8 (6.8) | 0.98 (0.43 – 2.26) | 0.91 (0.40 – 2.09) | 0.75 (0.32 – 1.76) | 19 (10.9) | 1.55 (0.84 – 2.87) | 1.52 (0.87 – 2.65) | 1.38 (0.76 – 2.51) |
| *Emergency CS* | 114 (9.4) | 17 (14.4) | 1.59 (0.92 – 2.78) | 1.57 (0.93 – 2.64) | 1.31 (0.74 – 2.33) | 20 (11.4) | 1.25 (0.73 – 2.13) | 1.24 (0.72 – 2.12) | 1.08 (0.62 – 1.86) |
| *Missing* | 25 (2.1) | 1 (0.8) |  |  |  | 1 (0.6) |  |  |  |
| *Child outcomes* |  |  |  |  |  |  |  |  |  |
| *Gestational age (weeks)* |  |  |  |  |  |  |  |  |  |
| *Mean* | 40.1 | 40.1 |  |  |  | 39.7 |  |  |  |
| *Median* | 40.4 | 40.2 |  |  |  | 40.1 |  |  |  |
| *z-score* |  |  | -0.01 (-0.21; 0.19) | 0.01 (-0.19; 0.21) | 0.02 (-0.18; 0.22) |  | -0.24 (-0.47; -0.01) | -0.23 (-0.44; -0.02) | -0.23 (-0.44; -0.03) |
| *Birth weight (grams)* |  |  |  |  |  |  |  |  |  |
| *Mean* | 3548 | 3604 |  |  |  | 3497 |  |  |  |
| *Median* | 3550 | 3645 |  |  |  | 3530 |  |  |  |
| *z-score* |  |  | 0.11 (-0.06; 0.28) | 0.11 (-0.09; 0.31) | 0.07 (-0.11; 0.26) |  | -0.10 (-0.28; 0.08) | -0.10 (-0.27; 0.08) | -0.14 (-0.35; 0.07) |
| *z-score BW by Gestational age* |  |  | 0.13 (-0.06; 0.31) | 0.12 (-0.07; 0.30) | 0.06 (-0.14; 0.26) |  | -0.01 (-0.18; 0.17) | -0.01 (-0.19; 0.17) | -0.07 (-0.24; 0.09) |
| *Respiratory distress**** | 51 (4.2) | 7 (5.9) | 1.44 (0.61 – 3.41) | 1.35 (0.57 – 3.20) | 1.53 (0.57 – 4.12) | 14 (8.0) | 1.99 (0.94 – 4.21) | 1.96 (1.05 – 3.64) | 1.76 (0.87 – 3.55) |

*Adjusted for stress (anxiety/depression)
**Adjusted for stress (anxiety/depression), maternal age, BMI
***ICD-10 diagnoses P22-P28 in the newborn child
