# Supplementary material for: Maternal asthma and the role of stress, sensitization, and lung function on pregnancy outcomes: MAESTRO cohort study
Source: J Allergy Clin Immunol Glob. 2026 Mar 19;5(3):100683. doi: 10.1016/j.jacig.2026.100683 (PMC13087686; doi:10.1016/j.jacig.2026.100683)
Supplement: Supplementary Table E4 [file mmc4.docx]

**Supplemental table 4.** Z-score for lung function; pre-Bronchodilation

|  | Z-score for FEV_1_ OR/Beta coefficients (95% CI) | | | | Z-score for FVC OR/Beta coefficients (95% CI) | | | | Z-score for FEV_1_/FVC OR/Beta coefficients (95% CI) | | | |
| --- | --- | --- | --- | --- | --- | --- | --- | --- | --- | --- | --- | --- |
| Exposure groups |  | **Crude** | **Adj*** | **Adj**** |  | **Crude** | **Adj*** | **Adj**** |  | **Crude** | **Adj*** | **Adj**** |
|  |  |  |  |  |  |  |  |  |  |  |  |  |
|  |  |  |  |  |  |  |  |  |  |  |  |  |
| *Maternal outcomes* |  |  |  |  |  |  |  |  |  |  |  |  |
| *Delivery Mode* |  |  |  |  |  |  |  |  |  |  |  |  |
| *Vaginal non-instrumental delivery* |  | Ref. | Ref. | Ref. |  | Ref. | Ref. | Ref. |  | Ref. | Ref. | Ref. |
| *Vaginal instrumental delivery* |  | 0.84 (0.49 – 1.44) | 0.78 (0.42 – 1.47) | 0.75 (0.38 – 1.50) |  | 0.80 (0.50 – 1.30) | 0.78 (0.46 – 1.34) | 0.76 (0.41 – 1.42) |  | 1.03 (0.56 – 1.90) | 0.95 (0.51 – 1.80) | 0.94 (0.49 – 1.80) |
| *Elective CS* |  | 0.91 (0.54 – 1.54) | 0.74 (0.41 – 1.35) | 0.72 (0.36 – 1.43) |  | 0.81 (0.41 – 1.59) | 0.75 (0.39 – 1.42) | 0.72 (0.38 – 1.37) |  | 1.24 (0.71 – 2.16) | 0.97 (0.53 – 1.80) | 0.96 (0.53 – 1.73) |
| *Emergency CS* |  | 0.90 (0.67 – 1.21) | 0.87 (0.55 – 1.38) | 0.87 (0.58 – 1.28) |  | 0.89 (0.58 – 1.37) | 0.88 (0.53 – 1.44) | 0.87 (0.50 – 1.50) |  | 1.11 (0.63 – 1.94) | 1.09 (0.62 – 1.92) | 1.09 (0.63 – 1.89) |
| *Missing* |  |  |  |  |  |  |  |  |  |  |  |  |
| *Child outcomes* |  |  |  |  |  |  |  |  |  |  |  |  |
| *Gestational age (weeks)* |  |  |  |  |  |  |  |  |  |  |  |  |
| *z-score* |  | 0.03 (-0.07; 0.12) | 0.02 (-0.10; 0.14) | 0.03 (-0.09; 0.14) |  | 0.03 (-0.12; 0.18) | 0.02 (-0.12; 0.15) | 0.02 (-0.13; 0.17) |  | -0.02 (-0.19; 0.14) | -0.01 (-0.17; 0.15) | -0.01 (-0.18; 0.17) |
| *Birth weight (grams)* |  |  |  |  |  |  |  |  |  |  |  |  |
| *z-score* |  | -0.02 (-0.15; 0.11) | -0.04 (-0.17; 0.08) | -0.04 (-0.16; 0.08) |  | -0.03 (-0.15; 0.09) | -0.06 (-0.19; 0.08) | -0.05 (-0.19; 0.08) |  | -0.02 (-0.17; 0.14) | -0.03 (-0.19; 0.14) | -0.03 (-0.20; 0.15) |
| *BW for gestational age* |  |  |  |  |  |  |  |  |  |  |  |  |
| *z-score* |  | -0.04 (-0.13; 0.06) | -0.07 (-0.19; 0.05) | -0.07 (-0.19; 0.06) |  | -0.06 (-0.19; 0.06) | -0.08 (-0.22; 0.05) | -0.08 (-0.22; 0.06) |  | 0.01 (-0.14; 0.15) | -0.02 (-0.17; 0.13) | -0.02 (-0.17; 0.13) |
| *Respiratory distress**** |  | 0.72 (0.37 – 1.45) | 0.71 (0.36 – 1.40) | 0.69 (0.38 – 1.24) |  | 0.76 (0.38 – 1.52) | 0.78 (0.39 – 1.58) | 0.76 (0.37 – 1.59) |  | 0.88 (0.45 – 1.71) | 0.81 (0.44 – 1.47) | 0.80 (0.44 – 1.46) |

*Adjusted for stress (anxiety/depression), and BMI
**Adjusted for stress (anxiety/depression), BMI, and education
*** ICD-10 diagnoses P22-P28 in the newborn child

**Supplemental table 4.** Z-score for lung function; post Bronchodilation

|  | Z-score for FEV_1_ OR/Beta coefficients (95% CI) | | | | Z-score for FVC OR/Beta coefficients (95% CI) | | | | Z-score for FEV_1_/FVC OR/Beta coefficients (95% CI) | | | |
| --- | --- | --- | --- | --- | --- | --- | --- | --- | --- | --- | --- | --- |
| Exposure groups |  | **Crude** | **Adj*** | **Adj**** |  | **Crude** | **Adj*** | **Adj**** |  | **Crude** | **Adj*** | **Adj**** |
|  |  |  |  |  |  |  |  |  |  |  |  |  |
|  |  |  |  |  |  |  |  |  |  |  |  |  |
| *Maternal outcomes* |  |  |  |  |  |  |  |  |  |  |  |  |
| *Delivery Mode* |  |  |  |  |  |  |  |  |  |  |  |  |
| *Vaginal non-instrumental delivery* |  | Ref. | Ref. | Ref. |  | Ref. | Ref. | Ref. |  | Ref. | Ref. | Ref. |
| *Vaginal instrumental delivery* |  | 1.22 (0.61 – 2.44) | 1.19 (0.69 – 2.05) | 1.16 (0.62 – 2.18) |  | 1.15 (0.56 – 2.37) | 1.11 (0.57 – 2.16) | 1.07 (0.43 – 2.67) |  | 1.43 (0.66 – 3.12) | 1.47 (0.54 – 4.02) | 1.45 (0.54 – 3.86) |
| *Elective CS* |  | 0.82 (0.46 – 1.46) | 0.75 (0.35 – 1.64) | 0.72 (0.32 – 1.63) |  | 0.79 (0.44 – 1.40) | 0.77 (0.39 – 1.53) | 0.74 (0.33 – 1.68) |  | 1.07 (0.69 – 1.67) | 0.90 (0.53 – 1.51) | 0.89 (0.55 – 1.44) |
| *Emergency CS* |  | 0.99 (0.61 – 1.59) | 0.94 (0.64 – 1.39) | 0.93 (0.61 – 1.43) |  | 0.93 (0.59 – 1.48) | 0.88 (0.45 – 1.73) | 0.87 (0.44 – 1.70) |  | 1.29 (0.64 – 2.56) | 1.31 (0.68 – 2.53) | 1.31 (0.71 – 2.41) |
| *Missing* |  |  |  |  |  |  |  |  |  |  |  |  |
| *Child outcomes* |  |  |  |  |  |  |  |  |  |  |  |  |
| *Gestational age (weeks)* |  |  |  |  |  |  |  |  |  |  |  |  |
| *z-score* |  | 0.03 (-0.08; 0.13) | 0.03 (-0.07; 0.12) | 0.03 (-0.08; 0.15) |  | 0.04 (-0.10; 0.18) | 0.03 (-0.12; 0.18) | 0.04 (-0.10; 0.18) |  | -0.04 (-0.20; 0.12) | -0.02 (-0.24; 0.20) | -0.02 (-0.22; 0.17) |
| *Birth weight (grams)* |  |  |  |  |  |  |  |  |  |  |  |  |
| *z-score* |  | -0.04 (-0.15; 0.08) | -0.05 (-0.19; 0.09) | -0.04 (-0.17; 0.08) |  | -0.04 (-0.18; 0.10) | -0.06 (-0.20; 0.09) | -0.05 (-0.17; 0.07) |  | -0.02 (-0.20; 0.16) | -0.01 (-0.22; 0.19) | -0.01 (-0.21; 0.19) |
| *BW for gestational age* |  |  |  |  |  |  |  |  |  |  |  |  |
| *z-score* |  | -0.06 (-0.19; 0.08) | -0.07 (-0.20; 0.06) | -0.07 (-0.22; 0.08) |  | -0.08 (-0.23; 0.07) | -0.09 (-0.23; 0.05) | -0.09 (-0.25; 0.07) |  | 0.01 (-0.15; 0.18) | 0.01 (-0.14; 0.16) | 0.01 (-0.15; 0.18) |
| *Respiratory distress**** |  | 0.80 (0.44 – 1.46) | 0.74 (0.36 – 1.52) | 0.72 (0.41 – 1.27) |  | 0.86 (0.48 – 1.53) | 0.82 (0.39 – 1.73) | 0.80 (0.46 – 1.42) |  | 0.82 (0.37 – 1.82) | 0.76 (0.34 – 1.69) | 0.75 (0.32 – 1.80) |

*Adjusted for stress (anxiety/depression), and BMI
**Adjusted for stress (anxiety/depression), BMI, and education
*** ICD-10 diagnoses P22-P28 in the newborn child
