# Supplementary material for: Maternal asthma and the role of stress, sensitization, and lung function on pregnancy outcomes: MAESTRO cohort study
Source: J Allergy Clin Immunol Glob. 2026 Mar 19;5(3):100683. doi: 10.1016/j.jacig.2026.100683 (PMC13087686; doi:10.1016/j.jacig.2026.100683)
Supplement: Supplementary Table E5 [file mmc5.docx]

**Supplemental table 5.** Non-allergic and allergic asthma and odds ratios and beta-coefficients for adverse pregnancy and perinatal outcomes. Data only from individuals that completed early pregnancy questionnaire.

|  | w/o asthma | Asthma, IgE negative | | | | | Asthma, IgE positive | | | | |
| --- | --- | --- | --- | --- | --- | --- | --- | --- | --- | --- | --- |
| Exposure groups |  | **n(%)** | **Crude OR/β-coeff** | **Adj* OR/β-coeff** | **Adj** OR/β-coeff** | **Adj**† **OR/β-coeff** | **n(%)** | **Crude OR/β-coeff** | **Adj* OR/β-coeff** | **Adj** OR/β-coeff** | **Adj**† **OR/β-coeff** |
|  | 860 | 89 | (95% CI) | (95% CI) | (95% CI) | (95% CI) | 131 | (95% CI) | (95% CI) | (95% CI) | (95% CI) |
|  |  |  |  |  |  |  |  |  |  |  |  |
| *Maternal outcomes* |  |  |  |  |  |  |  |  |  |  |  |
| *Hypertension in pregnancy* | 41 (4.8) | 5 (5.6) | 1.19 (0.43 – 3.26) | 1.11 (0.37 – 3.34) | 0.78 (0.24 – 2.51) | 0.80 (0.28 – 2.30) | 3 (2.3) | 0.47 (0.14 – 1.51) | 0.46 (0.15 – 1.36) | 0.44 (0.12 – 1.53) | 0.47 (0.12 – 1.82) |
| *Delivery Mode* |  |  |  |  |  |  |  |  |  |  |  |
| *Vaginal non-instrumental delivery* | 643 (74.7) | 65 (73.0) | Ref. | Ref. | Ref. | Ref. | 97 (74.1) | Ref. | Ref. | Ref. | Ref. |
| *Vaginal instrumental delivery* | 60 (7.0) | 6 (6.7) | 0.99 (0.42 – 2.34) | 0.95 (0.39 – 2.33) | 0.96 (0.33 – 2.77) | 0.99 (0.35 – 2.81) | 3 (2.3) | 0.33 (0.00 – 586) | 0.33 (0.00 – 42.9) | 0.35 (0.00 – 203) | 0.38 (0.13 – 1.10) |
| *Elective CS* | 59 (6.9) | 6 6.7) | 1.01 (0.37 – 2.71) | 0.93 (0.33 – 2.66) | 0.77 (0.29 – 2.08) | 0.80 (0.31 – 2.04) | 13 (9.9) | 1.46 (0.78 – 2.72) | 1.42 (0.69 – 2.93) | 1.35 (0.69 – 2.66) | 1.26 (0.66 – 2.41) |
| *Emergency CS* | 84 (9.8) | 11 (12.4) | 1.30 (0.59 – 2.84) | 1.26 (0.56 – 2.82) | 0.98 (0.45 – 2.13) | 1.01 (0.45 – 2.25) | 17 (13.0) | 1.34 (0.74 – 2.45) | 1.33 (0.69 – 2.54) | 1.14 (0.62 – 2.08) | 1.06 (0.53 – 2.12) |
| *Missing* | 14 (1.6) | 1 (1.5) |  |  |  |  | 1 (0.8) |  |  |  |  |
| *Child outcomes* |  |  |  |  |  |  |  |  |  |  |  |
| *Gestational age (weeks)* |  |  |  |  |  |  |  |  |  |  |  |
| *Mean* | 40.1 | 40.1 |  |  |  |  | 39.6 |  |  |  |  |
| *Median* | 40.3 | 40.3 |  |  |  |  | 40.0 |  |  |  |  |
| *z-score* |  |  | 0.00 (-0.24; 0.23) | 0.01 (-0.18; 0.20) | 0.02 (-0.22; 0.25) | 0.01 (-0.22; 0.23) |  | -0.28 (-0.53; -0.02) | -0.27 (-0.50; -0.04) | -0.25 (-0.49; -0.01) | -0.25 (-0.52; 0.02) |
| *Birth weight (grams)* |  |  |  |  |  |  |  |  |  |  |  |
| *Mean* | 3541 | 3605 |  |  |  |  | 3479 |  |  |  |  |
| *Median* | 3540 | 3630 |  |  |  |  | 3515 |  |  |  |  |
| *z-score* |  |  | 0.12 (-0.08; 0.33) | 0.12 (-0.10; 0.34) | 0.08 (-0.16; 0.32) | 0.06 (-0.15; 0.28) |  | -0.12 (-0.34; 0.11) | -0.12 (-0.33; 0.10) | -0.13 (-0.34; 0.07) | -0.13 (-0.37; 0.10) |
| *z-score BW by Gestational age* |  |  | 0.15 (-0.08; 0.39) | 0.14 (-0.06; 0.34) | 0.08 (-0.15; 0.30) | -0.01 (-0.23; 0.22) |  | -0.02 (-0.20; 0.15) | -0.03 (-0.25; 0.20) | -0.07 (-0.28; 0.14) | -0.07 (-0.29; 0.14) |
| *Respiratory distress* | 38 (4.4) | 5 (5.6) | 1.29 (0.43 – 3.89) | 1.21 (0.37 – 3.97) | 0.08 (0.40 – 4.58) | 0.07 (0.14 – 0.28) | 11 (8.4) | 1.98 (0.95 – 4.13) | 1.94 (0.87 – 4.34) | 1.54 (0.59 – 4.00) | 1.59 (0.66 – 3.81) |

*Adjusted for stress (anxiety/depression
**Adjusted for stress (anxiety/depression), maternal age, BMI
†Adjusted for stress (anxiety/depression), maternal age, BMI, maternal education
